# Analysis of the 1-year efficacy of four different surgical methods for treating Chinese super obese (BMI ≥ 50 kg/m^2^) patients

**DOI:** 10.1038/s41598-024-60983-x

**Published:** 2024-05-07

**Authors:** Zheng Zhang, Lun Wang, Zhiqiang Wei, Zhenhua Zhang, Liang Cui, Tao Jiang

**Affiliations:** Department of Bariatric and Metabolic Surgery, China-Japan Union Hospital, Jilin University, No. 126 Xiantai Avenue, Changchun, 130033 Jilin China

**Keywords:** Roux-en-Y gastric bypass, Sleeve gastrectomy, Single anastomosis duodenal-ileal bypass with sleeve gastrectomy, Biliopancreatic diversion with duodenal switch, Super obesity, Metabolic diseases, Laparoscopy, Gastrointestinal system, Endocrine system and metabolic diseases

## Abstract

This study aimed to retrospectively analyze the perioperative and postoperative follow-up data of patients with super obesity who had undergone RYGB, SG, BPD/DS, and SADI-S. A retrospective observational study was conducted to analyze the perioperative and postoperative follow-up data of 60 patients with super obesity who had undergone bariatric surgery. A total of 34 men and 26 women were included in this study. The participants had an average preoperative BMI of 53.81 ± 3.25 kg/m^2^. The body weight and BMI of all four patient groups decreased significantly at 3, 6, and 12 months postoperatively compared with the preoperative values. Additionally, the TWL (%) and EWL (%) of all four groups increased gradually over the same period. Compared with the preoperative values, the systolic and diastolic blood pressure, glycosylated hemoglobin, uric acid, triglycerides, and total cholesterol decreased to varying degrees in the four groups 1 year postoperatively. RYGB, SG, BPD/DS, and SADI-S are all safe and effective in treating super obese patients and improving their metabolic diseases to a certain extent.

## Introduction

With changes in lifestyle and dietary habits in the modern era, metabolic diseases, such as obesity, have become increasingly prevalent. The rise in obesity rates not only significantly impacts the quality of life of the affected individuals but also exerts an enormous strain on the healthcare system^1^. Super obesity, which is defined as having a body mass index (BMI) of ≥ 50 kg/m^2^, is the most severe form of obesity^2^. The population with super obesity has increased by 500% in the last decade and accounts for 30–50% of morbid obesity^3^. A study has estimated that super obesity can reduce a patient’s life expectancy by approximately 9.8 years^4^. Conservative treatment methods, such as diet control and exercise, have shown limited effectiveness in the treatment of these patients. Hence, metabolic surgery has emerged as the preferred option in managing patients with super obesity^5^. However, owing to the presence of underlying diseases prior to the surgery, the procedure can be challenging and the incidence of postoperative complications is higher than that in individuals with morbid obesity^6^. These factors may restrict the use of surgery to treat super obesity.

Currently, Roux-en-Y gastric bypass (RYGB) and sleeve gastrectomy (SG) are the most frequently performed metabolic surgeries worldwide (45.9% and 39.6%, respectively)^7^. Although RYGB appears to be advantageous for patients with super obesity, as the follow-up time increases, there is no difference in weight change or comorbidity relief between the two procedures^8–10^. Biliopancreatic diversion with duodenal switch (BPD/DS) is acclaimed as one of the most effective bariatric surgeries for obesity, and its curative effect on super obesity is significantly stronger than that of RYGB^11^. Single anastomosis duodenal–ileal bypass with sleeve gastrectomy (SADI-S) was proposed by Sanchez et al.^12^ based on the surgical ideas of BPD/DS. In spite of gaining popularity amidst bariatric and metabolic surgeons in recent years, there is a lack of comprehensive studies on its effectiveness in treating patients with super obesity.

To date, no clinical study in China has examined the effectiveness of multiple bariatric surgeries, including SADI-S, for treating super obesity. Hence, this study retrospectively analyzed the perioperative and postoperative follow-up data of four groups of patients with super obesity. The aim was to determine the efficacy of each surgery in this population and guide clinical treatment selection.

## Methods

### Patients

This study retrospectively analyzed patients with super obesity who underwent surgery at the bariatric center in a university-affiliated hospital between November 2015 and June 2022. All patients underwent physical and laboratory examinations as well as multidisciplinary consultations preoperatively. In addition, information on preoperative metabolic diseases, such as hypertension, type 2 diabetes, hyperuricemia, hyperlipidemia, and hypercholesterolemia, was collected. The patients were followed up at 3, 6, and 12 months postoperatively. Preoperative characteristics, intraoperative and postoperative conditions, weight loss effects, alterations in trace elements and nutrients, and relief from comorbidities were analyzed.

### Surgical techniques

All four surgeries were performed at the same bariatric center.

#### RYGB

The procedure involved creating a subcardial gastric pouch completely separated from the fundus. The jejunum was subsequently transected at 75 cm proximal from the ligament of Treitz. Using a 16-Fr gastric tube as a guide, the distal jejunum–gastric sac was anastomosed side-to-side. This step was followed by the anastomosis of the proximal end of the jejunum, with the jejunum at 100 cm below the gastrojejunal anastomosis. Each anastomosis and mesenteric hole was finally sutured.

#### SG

Initially, the gastrocolic ligament was opened, and the posterior gastric wall was freed completely. A 32-Fr bougie was inserted into the stomach, followed by using a straight cutting stapler to cut and suture the stomach wall from the great curvature, approximately 3 cm away from the pylorus. The stomach was then trimmed into a sleeve and intermittently sutured to the omentum.

#### BPD/DS

Measure 100 cm from the ileocecal junction to locate the common channel, and measure 200 cm from the same point to locate the digestive limb. Mark these points accordingly. The gastrocolic ligament was opened, and sleeve gastrectomy was performed under 36-Fr bougie. The duodenum was transected 2 cm distal to the pylorus, followed by an end-to-side anastomosis with the duodenal stump located 300 cm proximal to the ileocecal valve. The small intestine was then transected 2 cm proximal to the anastomosis, and an anastomosis between the biliopancreatic limbs and the ileum was performed 100 cm proximal to the ileocecal valve. Mesenteric holes were closed as per routine procedure, and finally, sutured the gastric resection edge and omentum.

#### SADI-S

Following sleeve gastrectomy with a 34-Fr bougie, the duodenum was severed 2 cm below the pylorus. Subsequently, an end-to-end anastomosis was created connecting the proximal duodenum and ileum, while maintaining a 300 cm common channel. The gastric resection edge and omentum were then sutured continuously.

### Outcomes and variables

Indicators related to surgical safety included the operation time, days of postoperative hospital stay, and postoperative complications. Postoperative complications were assessed using the Clavien-Dindo classification^13^. Weight parameters were defined as follows :

$$\begin{aligned} \% {\text{EWL }} & = \, \left[ {\left( {\text{initial weight}}  \, - \,  {\text{postoperative weight}} \right)/\left( {\text{initial weight}}  \, - \,  {\text{ideal weight}} \right)} \right] \, \times { 1}00\% , {\text{ideal weight }} & = {\text{ BMI of 23}}; \, \hfill \\ \% {\text{TWL }} & = \, \left[ {\left( {\text{initial weight}} \, - \, {\text{postoperative weight}}\right) \, / \,{\text{initial weight}}} \right] \, \times { 1}00\% \hfill \\ \end{aligned}$$

Remission criteria for obesity-induced metabolic disease were defined as follows: remission of hypertension: blood pressure < 140/90 mmHg without medication; remission of type 2 diabetes: HbA1c < 6.5% without medication; remission of hyperuricemia: uric acid < 428 μmol/L (for men), uric acid < 357 μmol/L (for women); remission of dyslipidemia: triglyceride < 1.7 mmol/L and total cholesterol < 5.7 mmol/L without medication.

Criteria for nutritional deficiencies were as follows: hemoglobin < 110 g/L, iron < 8.9 µmol/L, albumin < 35 g/L, total protein < 62 g/L, folate < 3.2 ng/mL, vitamin B12 < 180 pmol/L, calcium < 2.10 mmol/L, and zinc < 11.1 μmol/L.

### Statistical analysis

SPSS 26.0 was used for statistical analysis. Measured data that adhere to a normal distribution are typically represented as mean ± standard deviation (Mean ± SD). One-way analysis of variance was used for comparisons between multiple groups, and Bonferroni method was used to correct *P* values (P*) for post hoc pairwise comparisons (P* < 0.0083 was considered statistically significant). Paired t-test was used to compare data before and after surgery within the same group. Categorical data were analyzed using the chi-square test, and Fisher’s precision probability test was used to examine categorical data that did not meet the chi-square test criteria. *P* < 0.05 was considered statistically significant.

### Ethics approval

All procedures performed in studies involving human participants were in accordance with the ethical standards of the institutional and/or national research committee and with the 1964 Helsinki declaration and its later amendments or comparable ethical standards. This research including all experimental methods was approved by the ethics committee of China-Japan Union Hospital.

### Informed consent

Informed consent was obtained from all individual participants included in the study.

## Results

### Baseline characteristics

A total of 60 patients underwent the four procedures. According to the surgical methods, the patients were categorized into RYGB group (n = 10), SG group (n = 22), BPD/DS group (n = 14), and SADI-S group (n = 14). There were 34 men and 26 women in the entire group, with an average age of 31.30 ± 8.22 years. Their preoperative average weight and average BMI were 161.30 ± 16.73 kg and 53.81 ± 3.25 kg/m^2^, respectively. The baseline characteristics of the four groups of patients were comparable (Table 1).

**Table 1:** Patient characteristics before surgery.

Factor	RYGB(n = 10)	SG(n = 22)	BPD/DS(n = 14)	SADI-S(n = 14)	F/χ^2^	P
Gender (male/female), n	5/5	14/8	9/5	6/8	2.034	0.565
Mean age (years)	34.20 ± 10.16	31.55 ± 8.66	33.07 ± 7.07	27.07 ± 5.88	1.967	0.129
Preoperative body weight (kg)	161.75 ± 18.8	162.16 ± 16.78	161.3 ± 16.41	159.61 ± 17.21	0.066	0.978
Preoperative BMI (kg/m^2^)	55.20 ± 3.42	52.87 ± 2.63	54.76 ± 3.61	53.34 ± 3.36	1.792	0.159
HbA1c (%)	7.16 ± 1.86	6.66 ± 1.88	6.94 ± 1.08	6.24 ± 1.30	0.681	0.568
Systolic blood pressure (mmHg)	160.00 ± 29.20	153.77 ± 18.24	154.86 ± 36.55	149.50 ± 13.94	0.354	0.787
Diastolic blood pressure (mmHg)	90.30 ± 22.29	90.45 ± 13.02	90.79 ± 17.03	89.64 ± 15.53	0.012	0.998
Uric acid (μmol/L)	457.70 ± 109.17	460.24 ± 99.19	506.60 ± 157.42	531.51 ± 106.98	1.171	0.331
Triglyceride (mmol/L)	2.47 ± 1.57	2.23 ± 1.62	2.03 ± 0.58	1.69 ± 0.61	0.804	0.497
Total cholesterol (mmol/L)	4.73 ± 1.22	4.91 ± 0.90	5.65 ± 0.96	5.51 ± 1.56	2.096	0.112
With type 2 diabetes, n	5 (10)	5 (17)	7 (13)	4 (13)	2.716	0.437
With hypertension, n	6 (10)	17 (22)	9 (14)	11 (14)	1.822*	0.680
With hyperuricemia, n	7 (10)	15 (21)	8 (12)	11 (12)	2.610*	0.497
With hyperlipidemia, n	6 (10)	12 (22)	10 (14)	4 (12)	3.908	0.272
With hypercholesterolemia, n	2 (10)	3 (22)	6 (14)	3 (12)	3.923*	0.275

*Fisher's precision probability test used wherever appropriate.

### Perioperative outcomes

All procedures were performed laparoscopically, and there were no instances of conversion to laparotomy or mortality. Furthermore, the amount of blood loss during the surgery was minimal, and < 50 mL was recorded. The SG group exhibited significantly shorter surgical time than the other three groups (all P* < 0.0083), whereas the BPD/DS group displayed longer operative time than the other three groups, and this difference was statistically significant (all P* = 0.0000). However, there was no difference in postoperative hospital stay among the four groups (P* > 0.0083). In the RYGB group, two patients experienced postoperative complications, resulting in a complication rate of 20% (2/10). These complications, classified as Clavien-Dindo grade II, included instances of bleeding and anastomotic leakage. Following conservative treatment, all patients were discharged. In contrast, one patient in the BPD group encountered small intestinal obstruction post-surgery, categorized as Clavien-Dindo grade IIIb. The complication rate for this group was 7.14% (1/14). After undergoing reoperation, the patient was successfully was cured and discharged, as shown in Table 2.

**Table 2:** Perioperative parameters of RYGB, SG, BPD/DS, and SADI-S.

Factor	RYGB (n = 10)	SG (n = 22)	BPD/DS (n = 14)	SADI-S (n = 14)	F	P
Operation time (min)	202.22 ± 47.97	133.44 ± 35.15	283.07 ± 45.06	206.08 ± 25.60	37.590	0.000
Length of hospital stay (day)	10.67 ± 5.87	10.81 ± 4.31	8.00 ± 3.49	8.38 ± 2.99	1.702	0.179
Complications, n	2	0	1	0	–	–
Complication rate (%)	20	0	7.14	0	–	–

### Weight loss outcome

**Figure 1:**
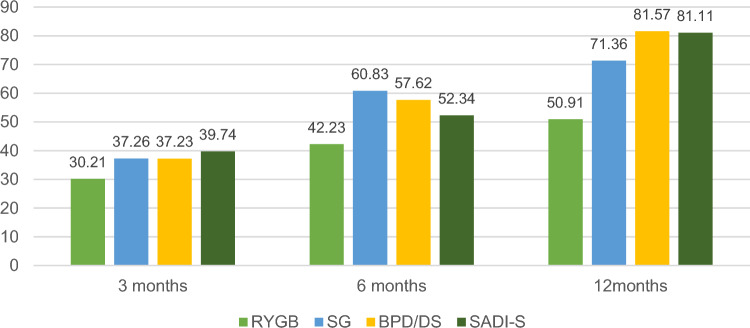
Changes in %EWL at 3, 6, and 12 months in different surgical groups.

**Figure 2:**
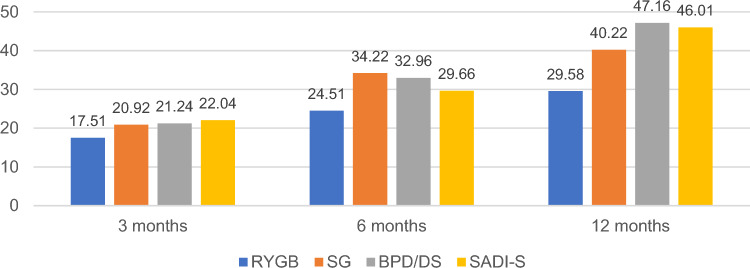
Changes in %TWL at 3, 6, and 12 months in different surgical groups.

Weight changes are shown in Table 3, Figs. 1 and 2. At 3 months postoperatively, pairwise comparisons revealed no significant differences in weight-related indicators across all four groups (all P* > 0.0083). At 6 months postoperatively, the SG group exhibited a significantly lower BMI compared to the RYGB group (P* = 0.0008). Additionally, the %EWL and %TWL outcomes for the SG and BPD/DS groups were notably superior to those of the RYGB group (P* = 0.0005, P* = 0.0007; P* = 0.0060, P* = 0.0053), with no significant variances observed among the remaining groups (all P* > 0.0083). At 12 months post-surgery, the weight loss-related indicators for the SG, BPD/DS, and SADI-S groups surpassed those of the RYGB group (all P* < 0.0083). Moreover, no significant distinctions were found between the SG, BPD/DS, and SADI-S groups (P* > 0.0083).

**Table 3:** Weight loss-related outcomes in the RYGB, SG, BPD/DS, and SADI-S.

	RYGB (n = 10)	SG (n = 22)	BPD/DS (n = 14)	SADI-S (n = 14)	F	P
3 months	10 (100%)	21 (95.5%)	11 (78.6%)	9 (64.3%)	–	–
Weight (kg)	133.30 ± 15.80	128.11 ± 18.33	122.91 ± 10.40	124.39 ± 17.55	0.829	0.485
BMI (kg/m^2^)	45.56 ± 3.88	41.83 ± 4.04	42.70 ± 4.59	40.64 ± 3.77	2.674	0.058
%EWL	30.21 ± 6.66	37.26 ± 10.10	37.23 ± 8.02	39.74 ± 7.77	2.222	0.098
%TWL	17.51 ± 3.62	20.92 ± 5.39	21.24 ± 4.33	22.04 ± 3.88	1.873	0.147
6 months	10 (100%)	18 (81.8%)	12 (85.7%)	13 (92.9%)	–	–
Weight (kg)	121.95 ± 13.34	106.58 ± 17.63	105.11 ± 11.08	112.74 ± 17.46	2.746	0.053
BMI (kg/m^2^)	41.69 ± 3.23	34.92 ± 5.15	36.51 ± 5.60	37.65 ± 4.46	4.383	0.008
%EWL	42.23 ± 5.56	60.83 ± 14.49	57.62 ± 13.14	52.34 ± 12.74	5.104	0.004
%TWL	24.51 ± 2.83	34.22 ± 7.82	32.96 ± 7.17	29.66 ± 6.85	4.935	0.005
12 months	10 (100%)	19 (86.4%)	12 (85.7%)	13 (92.9%)	–	–
Weight (kg)	113.80 ± 15.47	95.31 ± 15.58	83.21 ± 12.24	86.42 ± 14.42	9.525	0.000
BMI (kg/m^2^)	38.87 ± 3.87	31.67 ± 5.16	28.75 ± 3.75	28.90 ± 4.28	12.126	0.000
%EWL	50.91 ± 10.55	71.36 ± 15.59	81.57 ± 12.58	81.11 ± 12.77	12.175	0.000
%TWL	29.58 ± 5.88	40.22 ± 8.79	47.16 ± 7.96	46.01 ± 6.97	11.709	0.000

### Comorbidity improvement and resolution

At 12 months postoperatively, hypertension, type 2 diabetes mellitus, hyperuricemia, blood lipid levels, and other related indexes were decreased in the RYGB group, but the difference was not statistically significant (*P* > 0.05). Furthermore, there was an improvement in hypertension and hyperuricemia in the SG group compared with the preoperative values (*P* < 0.05). Of the blood lipid indexes, triglyceride levels were improved significantly (*P* < 0.05), but there was no significant change in the total cholesterol level (*P* > 0.05). Type 2 diabetes showed improvement postoperatively, but the difference was not statistically significant (*P* > 0.05). In the BPD/DS group, hypertension, type 2 diabetes, hyperuricemia, blood lipid levels, and other related indexes were decreased significantly (*P* < 0.05). Glycated hemoglobin was decreased in SADI-S group, but the difference was not statistically significant (*P* > 0.05), and other related metabolic indexes were significantly improved (*P* < 0.05), as shown in Table 4.

**Table 4:** Comparison of remission of metabolic diseases related to SG, RYGB, BPD, and SADI at 12 months after and before the operation.

Factor	RYGB			SG			BPD/DS			SADI-S		
	12 months	t	P	12 months	t	P	12 months	t	P	12 months	t	P
HbA1c (%)	5.53 ± 0.41	1.699	0.115	5.14 ± 0.29	1.766	0.093	4.64 ± 0.26	7.484	0.000	4.833 ± 0.40	1.944	0.072
SBP (mmHg)	129.00 ± 19.44	1.934	0.077	127.29 ± 12.58	2.195	0.035	120.00 ± 17.88	2.649	0.015	109.85 ± 14.78	6.025	0.000
DBP (mmHg)	75.25 ± 8.22	1.289	0.222	81.29 ± 10.79	4.751	0.000	73.33 ± 11.93	2.672	0.014	64.43 ± 9.55	3.911	0.001
Uric acid (μmol/L)	389.40 ± 61.79	1.545	0.147	379.80 ± 93.26	2.628	0.013	352.81 ± 72.23	2.591	0.020	373.68 ± 76.06	3.760	0.001
Triglyceride (mmol/L)	1.28 ± 0.41	1.469	0.168	1.03 ± 0.38	3..310	0.003	1.19 ± 0.36	3.882	0.001	1.04 ± 0.75	2.166	0.043
Total cholesterol (mmol/L)	4.17 ± 0.58	0.858	0.408	4.92 ± 0.84	− 0.053	0.958	3.50 ± 0.23	8.050	0.000	3.66 ± 0.79	3.543	0.02

### Nutritional outcomes

Nutritional indicators of the four groups of patients 12 months postoperatively were compared with the preoperative values, as presented in Table 5. The RYGB group showed no changes in the relevant indexes compared with the preoperative values (*P* > 0.05). The SG group showed a decrease in vitamin B12 and a significant increase in serum iron compared with the preoperative levels (*P* = 0.005; *P* = 0.001). The BPD/DS group exhibited significantly lower levels of hemoglobin, serum iron, and serum zinc than the preoperative levels (*P* = 0.035; *P* = 0.018; *P* = 0.001). In the SADI-S group, there were no significant differences in other indexes, except for a decrease in serum zinc compared with the preoperative level (*P* = 0.004).

**Table 5:** Comparison of the nutritional indicators of SG, RYGB, SADI, and BPD-DS at 1 year after and before the operation.

	Total protein (g/L)	Albumin (g/L)	Hemoglobin (g/L)	Folate (ng/mL)	Vitamin B12 (pmol/L)	Calcium (mmol/L)	Iron (µmol/L)	Zinc (µmol/L)
Normal range	62–83	35–52	110–150	> 3.2	180–916	2.10–2.55	8.9–32.3	11.1–19.5
RYGB								
Baseline	74.00 ± 5.87	42.50 ± 3.03	143.50 ± 25.16	20.14 ± 12.66	280.11 ± 73.34	2.28 ± 0.10	14.56 ± 7.74	13.59 ± 3.89
12 months	72.20 ± 1.30	42.14 ± 1.28	140.20 ± 15.94	30.20 ± 13.30	219.22 ± 81.59	2.30 ± 0.06	20.20 ± 8.63	13.44 ± 2.94
t	0.925	0.251	0.265	−1.070	1.433	− 3.65	− 0.858	0.060
P	0.375	0.806	0.795	0.326	0.177	0.722	0.517	0.953
SG								
Baseline	71.14 ± 3.43	42.21 ± 2.38	152.70 ± 17.03	15.78 ± 8.68	370.18 ± 119.37	2.26 ± 0.08	13.86 ± 5.56	14.11 ± 2.20
12 months	70.24 ± 4.79	41.01 ± 2.37	141.00 ± 17.13	15.30 ± 8.92	251.20 ± 107.15	2.31 ± 0.11	22.27 ± 6.14	13.42 ± 1.38
t	0.648	1.442	1.967	0.157	2.980	− 1.638	− 3.810	0.913
P	0.522	0.159	0.058	0.876	0.005	0.111	0.001	0.369
BPD/DS								
Baseline	72.17 ± 5.13	41.70 ± 3.43	150.00 ± 11.39	9.16 ± 4.06	311.37 ± 105.80	2.33 ± 0.15	17.01 ± 4.67	14.72 ± 1.86
12 months	67.32 ± 8.80	37.74 ± 8.87	130.70 ± 23.73	8.76 ± 4.94	414.56 ± 432.66	2.29 ± 0.20	11.92 ± 4.82	10.63 ± 3.01
t	1.554	1.336	2.370	0.214	− 0.737	0.454	2.556	4.017
P	0.143	0.208	0.035	0.833	0.478	0.655	0.018	0.001
SADI-S								
Baseline	73.79 ± 6.31	40.08 ± 4.05	143.69 ± 17.90	13.87 ± 8.01	349.89 ± 146.16	2.38 ± 0.14	18.31 ± 10.37	15.10 ± 3.21
12 months	68.37 ± 7.49	38.92 ± 5.66	135.78 ± 16.36	7.39 ± 6.73	577.48 ± 385.64	2.35 ± 0.11	13.39 ± 4.59	11.24 ± 1.43
t	1.711	0.520	1.016	1.897	− 1.737	0.502	1.308	3.321
P	0.105	0.610	0.323	0.075	0.100	0.622	0.208	0.004

## Discussion

Over the last 50 years, bariatric and metabolic surgeons worldwide have collaborated to develop various surgical procedures to address obesity and related metabolic diseases. As a result, surgery has become the major treatment option.

A 2016 global survey by The International Federation for the Surgery of Obesity and Metabolic Disorders found^14^ that the most popular surgical procedures in the world are RYGB, SG, and BPD/DS. For patients with a BMI < 50 kg/m^2^, various surgical methods present their own advantages and disadvantages. BPD/DS offers the highest weight loss and diabetes remission rates, but it is also the most complex procedure with a risk of post-surgery malnutrition complications^15^. RYGB and SG are currently the most popular surgical options. RYGB provides effective long-term weight loss and hypoglycemic benefits, being relatively easier to perform compared to BPD-DS. However, there is a potential risk of malignancy in the remaining stomach post-surgery, particularly at the anastomosis site. Postoperative complications may include ulcers, dumping syndrome, and internal hernias^16^. LSG is gaining popularity due to its simplicity, low complication rates, and significant short- to medium-term effects. The same situation occurs in superobese patients, the discussion regarding the efficacy of surgical intervention and which weight loss surgery is most appropriate for this population is ongoing.

In China, there are few studies on surgery for patients with super obesity. Performing bariatric surgery on such patients is a technically challenging task owing to their thicker abdominal walls and higher accumulation of visceral fat, which makes the surgery more difficult than that in patients with morbid obesity (BMI of 35–49 kg/m^2^). Additionally, preoperative metabolic diseases may be more serious in patients with super obesity and could potentially result in increased perioperative morbidity and mortality^17,18^. An investigation has observed that super obesity is an independent risk factor for severe perioperative complications and the largest independent risk factor for 30-day postoperative mortality^19^. Therefore, a safe and effective way to treat patients with super obesity should be identified.

Kakarla et al.^6^ performed a study on the safety of surgery in individuals with super obesity and noted that the 30-day postoperative complication rate, mortality rate, and length of hospital stay were acceptable. Our study yielded similar results, with only 3 of the 60 patients experiencing complications. These patients too were successfully treated with conservative approaches or reoperation and were ultimately discharged. Strain et al.^20^ observed that the weight loss effect of metabolic surgery in patients with super obesity is comparable to that in patients with morbid obesity. After 2 years of follow-up, the %EWL was 63.4% in the super obesity group and 65.1% in the morbid obesity group. The results were comparable between the two groups (*P* = 0.64). Based on these findings, bariatric surgery can be concluded to be safe and beneficial for patients with super obesity.

In the surgical treatment of super obesity, the choice of the surgical method and the evaluation of its efficacy are of utmost importance. RYGB, which is one of the most prevalent bariatric procedures performed globally, has been shown to have long-lasting effects on weight control and improved metabolic status in patients with obesity^21^. Another study conducted a 10-year follow-up of patients with super obesity who had undergone RYGB treatment. The results signified that the comorbidities were significantly improved in all patients, and those with super obesity achieved a weight loss similar to that of patients with morbid obesity^22^. A meta-analysis found^10^ that 1-year %TWL and %EWL after RYGB were 36.06% and 59.73%, respectively. In our study, the 1-year %TWL and %EWL of the RYGB group were 29.58% and 50.91%, respectively. Despite these results, the RYGB group faced certain challenges, such as higher incidence of perioperative complications compared with other procedures and unsatisfactory remission of metabolic diseases.

As one of the available procedures for weight loss and improvement of metabolic diseases, SG was originally used as a “bridge” in the surgical treatment of super obesity^23^. However, owing to its effective weight loss results, SG became increasingly popular worldwide as an independent procedure. A study^24^ has demonstrated that SG offers significant benefits for patients with super obesity, particularly in terms of weight loss. In this research, the %EWL and %TWL at 1 year postoperatively in the SG group were 71.36% and 40.22%, respectively. This procedure presents several advantages over RYGB, including ease of implementation, especially in patients with super obesity. Moreover, SG is associated with fewer short-term complications and shorter operative time. Our study found that SG achieved better weight loss than RYGB 1 year after the treatment. However, these findings contradict the results of previous studies^8,9,25,26^. The successful outcomes achieved in our study may be attributed to use a small bougie tube.

According to the findings, of all procedures, BPD/DS with SADI-S provided the best weight loss outcomes for patients with super obesity. Marou et al.^27^ analyzed the total weight loss effect in 537 patients with super obesity at 6, 12, 24, and 60 months postoperatively. At 12 months, the BPD/DS group demonstrated the highest %TWL of 35.6%, followed by the RYGB (31.9%) and SG (27.3%) groups. These results agree with the findings of our study. The weight loss effect of BPD-DS (%EWL = 81.57%; %TWL = 47.16%) was significantly better than those of RYGB at 1 year postoperatively. It is also better than SG, but the difference is not significant. Similarly, Skogar^28^ also suggested that BPD/DS resulted in better weight loss and metabolic disease remission. In terms of surgical safety, a study has observed that although the operation time of BPD/DS is considerably longer than that of the other three groups, it is generally safe for patients with super obesity, which is in line with the outcomes of previous studies^29^. Our study indicated that nutritional deficiencies were more prevalent after BPD/DS surgery. As a result, a stronger nutritional supplementation plan was implemented for these patients postoperatively to achieve better long-term outcomes.

SADI-S is a simplified procedure for BPD-DS, and there are few reports on its long-term efficacy in patients with super obesity. In this study, SAID-S showed obvious advantages in terms of weight loss effect, improvement of metabolic diseases, and lower incidence of complications. There were no differences in %EWL or %TWL between the two groups compared with BPD/DS although %EWL and %TWL were slightly higher in BPD/DS (%EWL = 81.57%; %TWL = 47.16%) than in SADI-S (%EWL = 81.11%; %TWL = 46.01%). Moreover, the SADI-S technique was simple and the operation time was short, and its operation time is significantly lower than that of the BPD/DS group (P* = 0.0000). The study of Pereira et al.^30^ also obtained similar results. Enochs et al.^31^ conducted a retrospective analysis of 878 patients who underwent SADI-S, RYGB, and SG, including super obese patients. The results indicated that after two years, SADI-S, RYGB, and SG led to a decrease in BMI point of 19.5, 16.1, and 16.1 respectively. Additionally, SADI-S showed significantly better diabetes relief compared to the other two surgeries. Benjamin et al.'s study^32^ revealed a higher incidence of Clavien-Dindo grade II, IVa, and IVb complications in SADI-S compared to SG. However, in our study, no complications were observed in either group. It is worth noting that the operation time for SADI-S was significantly longer than that for SG (P* < 0.0083), likely due to the additional anastomosis involved in SADI-S. Thus, SADI-S has immense potential in the weight loss of individuals with super obesity and in the treatment of related metabolic diseases. However, its safety and long-term weight loss effect still require long-term observation.

Nonetheless, this study has some limitations. This is a single-center, short-term analysis study that compares the efficacies of four techniques in terms of postoperative weight loss. However, to validate the results, a multicenter, long-term study with a larger sample size is required. Additionally, incomplete follow-up data owing to few patients completing the follow-up may limit the findings of this study. Moreover, the limited number of patients in each group restricted the comparison of complication rates, comorbidity remission rates, and nutrient deficiency rates.

In conclusion, RYGB, SG, BPD/DS, and SADI-S are all safe and effective in treating super obese patients and improving their metabolic diseases to a certain extent. However, further research is needed to assess their long-term safety and effectiveness as a reference for metabolic surgical treatment of super-obese patients.

## Data Availability

The original contributions presented in the study are included in the article/Supplementary Material, further inquiries can be directed to the corresponding author.
